# Reversal of Glial and Neurovascular Markers of Unhealthy Brain Aging by Exercise in Middle-Aged Female Mice

**DOI:** 10.1371/journal.pone.0026812

**Published:** 2011-10-25

**Authors:** Caitlin S. Latimer, James L. Searcy, Michael T. Bridges, Lawrence D. Brewer, Jelena Popović, Eric M. Blalock, Philip W. Landfield, Olivier Thibault, Nada M. Porter

**Affiliations:** Department of Molecular and Biomedical Pharmacology, University of Kentucky College of Medicine, Lexington, Kentucky, United States of America; Centre National de la Recherche Scientifique - University of Bordeaux, France

## Abstract

Healthy brain aging and cognitive function are promoted by exercise. The benefits of exercise are attributed to several mechanisms, many which highlight its neuroprotective role via actions that enhance neurogenesis, neuronal morphology and/or neurotrophin release. However, the brain is also composed of glial and vascular elements, and comparatively less is known regarding the effects of exercise on these components in the aging brain. Here, we show that aerobic exercise at mid-age decreased markers of unhealthy brain aging including astrocyte hypertrophy, a hallmark of brain aging. Middle-aged female mice were assigned to a sedentary group or provided a running wheel for six weeks. Exercise decreased hippocampal astrocyte and myelin markers of aging but increased VEGF, a marker of angiogenesis. Brain vascular casts revealed exercise-induced structural modifications associated with improved endothelial function in the periphery. Our results suggest that age-related astrocyte hypertrophy/reactivity and myelin dysregulation are aggravated by a sedentary lifestyle and accompanying reductions in vascular function. However, these effects appear reversible with exercise initiated at mid-age. As this period of the lifespan coincides with the appearance of multiple markers of brain aging, including initial signs of cognitive decline, it may represent a window of opportunity for intervention as the brain appears to still possess significant vascular plasticity. These results may also have particular implications for aging females who are more susceptible than males to certain risk factors which contribute to vascular aging.

## Introduction

Exercise has been shown to be beneficial for cognitive function in aging [1]–[8]. Regular exercise is associated with various physiological and structural changes in the brain, especially in the hippocampus [2], [9], [7], an area that plays a key role in learning and memory [10]–[12]. Animal and human studies have highlighted several potential changes in neuronal function by which exercise may promote healthy brain aging including increases in neurotrophic factors, neurogenesis and neuronal plasticity [1], [3], [13]. Nevertheless, the effects of chronic exercise on age related changes in glial and cerebrovascular processes are relatively unexplored.

The brain parenchyma is composed of many cell types, but glia, are by far the most numerous [14]. It has long been appreciated that astroglial cells are involved in the inflammatory response of the aged brain and that an increase in astrocyte hypertrophy/reactivity is a consistent marker of brain aging across multiple species [15]–[22]. In addition, the process of myelination, mediated by oligodendroglial cells, is apparently dysregulated with aging [23]. Perhaps surprisingly, a number of studies indicate that activation of myelin-related genes/proteins and actual myelination are increased with brain aging [24], [23], [25]–[28]. Because glial processes regulate many aspects of neuronal function, these changes may have broad implications for the cognitive decline typical of unhealthy brain aging.

Interactions of these glial components of the parenchyma with cerebral blood vessels are also likely to play a critical role in brain aging. With aging, there is a decrease in vascularity and endothelial function which, in turn can affect cerebral perfusion pressure and hemodynamics [29]. Because the brain is so highly vascularized and depends on constant and sufficient cerebral blood flow [30], the impact of aging on brain function may depend on the extent to which such changes in the cerebrovasculature occur [29], [31]. Further, exercise and the vasoprotection it imparts may play a major role in modifying the extent of brain aging.

At midlife, low levels of physical activity in humans are already considered a risk factor for unhealthy brain aging [32]. On the other hand, exercise at this point in the lifespan appears to have significant effects on vascular function [33] and, thus, this period appears to represent an age at which significant vascular plasticity is still present. The beneficial effect of exercise may be particularly relevant for aging females as results from the Framingham and Whitehall cohorts show that some vascular risk factors (e.g., hypertension) may have a greater negative impact in women than men [34], [35]. Further, aerobic exercise, which positively impacts vascular health [36], [3], [29], [37], appears to confer greater cognitive benefits to aging women [2], [38], [6]. Women at midlife also experience hormonal changes, which along with physical inactivity, may further increase their vulnerability to certain aspects of vascular and brain aging [39]–[42].

Interestingly, this period of the lifespan in experimental animal models also coincides with the increased expression of many markers of brain aging, in particular an increase in astrocyte hypertrophy/reactivity and myelin-related changes [24], [43], [27]. Although, extensive work has been performed to examine the interactions of exercise with cognitive and neuronal function in aging, very little is known about the effects of exercise on midlife changes in vascular and glial biomarkers of brain aging. As these processes are intimately connected (i.e. astrocytic endfeet directly appose blood vessels in the brain and also appear to activate myelination processes) [44]–[47], it could well be that glial and vascular aging changes are of major importance to the cognitive and neuronal changes seen with aging. Therefore, the present studies were undertaken to test the hypothesis that exercise initiated at mid-age can slow the development of hippocampal glial and vascular biomarkers of early aging. Because of apparent selective effects of exercise on vascular and cognitive function in aging women [34], [2], [38], [35], [6], we studied the effects of exercise intervention in a middle-aged female animal model. Our results implicate glial and vascular changes as potential factors contributing to the beneficial effects of exercise on brain aging.

## Materials and Methods

### Ethics Statement

All procedures were in accordance with the National Research Council's Guide for the Care and Use of Laboratory Animals and an approved protocol (00770M) by the Institutional Animal Care and Use Committee (PHS Assurance #A3336-01) of the University of Kentucky's Office of Research Integrity.

### Animals/Exercise Protocol

Twenty middle-aged (11–13 months) female C57 BL/6 mice were obtained from the U.S. National Institute on Aging rodent colony. Upon arrival, animals were singly housed in a temperature and humidity controlled room in our AAALAC accredited animal care facility at the University of Kentucky Medical Center. All animals were maintained under standard housing conditions on a 12∶12 light/dark cycle and given free access to food and water. Animals were also handled and weighed three times/week throughout the study. Mice were allowed to recover from transportation for two weeks and then moved to a light-controlled environmental chamber containing standard (sedentary) or running wheel (exercised) cages (n = 10/group) for six weeks. No differences in initial body weight were present between groups. Because standard cages lacked a fixed wheel, we attempted to minimize the potential contribution of novelty provided by the presence of wheels for the exercised group by arranging cages with and without wheels in an alternating manner within the chamber. Thus, this arrangement may have provided some degree of novelty even for the sedentary animals in standard cages lacking wheels. Wheel activity was monitored throughout using ClockLab (Actimetrics). A separate group of young (4–6 months), middle-aged (12 months) and aged (22 months) female C57 BL/6 mice (n = 6–7/age) was used to assess age-related changes in the neurovasculature with vascular corrosion casting *(see below)*.

### Blood Pressure (BP)

After five weeks of wheel running, BP was measured noninvasively on restrained, conscious mice using the Visitech tail cuff system in the University of Kentucky Blood Pressure Research Core. To acclimate mice, habituation trials were conducted for two days prior to three days of data acquisition. Each day, the first ten measurements represented acclimation and the subsequent ten measurements were used for data analysis. Measures occurred at the same time each day.

### Tissue preparation

Following six weeks of running, animals were deeply anesthetized with sodium pentobarbital (100 mg/kg, i.p.) and perfused transcardially with cold saline using a peristaltic pump (4 ml/min). Brains were removed and divided along the longitudinal fissure. The right hemisphere was placed in 4% paraformaldehyde overnight and then transferred to 15% sucrose for cryoprotection. The left hemisphere was used for measures of vascular endothelial growth factor (VEGF).

### Immunohistochemistry

Astrocyte and myelin staining, in 30-µm-thick sections, were examined using antibodies against glial fibrillary acidic protein (rabbit GFAP; Abcam ab7779) and myelin basic protein (rat MBP; Abcam ab7349), respectively. Sections were bathed in Tris-buffered saline/0.5% Triton X-100 (TBS-T) containing primary antibody (1∶1000) for 18 h at 22°C. Sections were rinsed 3x in TBS-T and transferred to the appropriate biotinylated secondary antibody solution (1∶500) for 2 h (goat anti-rabbit, Chemicon AP132B; rabbit anti-rat, Abcam ab6733). Sections were then transferred to Vectastain Elite ABC reagent for 5 min (Vector) rinsed three times in TBS-T and incubated for 3 min with diaminobenzidine chromagen solution. Sections were processed in parallel.

Digitized images of stained sections were obtained using a Nikon Eclipse microscope with Nuance software (CRI) and images converted to gray scale. All images were acquired in a single session with the same camera settings. Semiquantitative analysis of chromagen staining (optical density) was determined using ImageJ (W. Rasband, NIH). Each data point represents the average optical density in the stratum radiatum of the CA1 region from three sections/animal. Background staining was accounted for by removing the on-tissue background optical density from each section. Analysis was performed on raw images and contrast enhancement was done for presentation purposes only.

### VEGF Analysis

VEGF concentrations were determined from hippocampal and cortical tissue homogenates by ELISA (R&D Systems). Brain tissue was homogenized in 1X PBS and subjected to freeze-thaw cycles to break membranes. Protein levels were determined via a Bradford analysis.

### Vascular Casting

Brain vascular casts were obtained according to Krucker *et al.* (2006). After being deeply anesthetized with sodium pentobarbital, animals were transcardially perfused for 5 min with 0.9% heparinized saline (25,000 U heparin/L), followed by a brief 1 min perfusion with 4% paraformaldehyde (10 ml/min-peristaltic). The vasculature was then perfused with 20 ml of Pu4ii polyurethane resin (VasQTec, Switzerland) using a syringe pump (4 ml/min). To promote cerebral perfusion, the descending aorta was ligated. The resin cured for at least 48 h at 22°C before brains were removed and incubated (50°C) in 7.5% KOH for 48 h followed by 48 h in 5% formic acid to macerate soft tissue. Casts were rinsed and stored in dH2O (−80°C) until lyophilization for 18–24 h. Casts were prepared for scanning electron microscopy and imaged using a S–3200-N Hitachi scanning electron microscope**.**

Scanning electron micrographs of the middle cerebral artery (MCA), identified by its size and location, were acquired. Arteries were easily distinguished from the more flaccid appearing veins. Additionally, impressions made by arterial endothelial cell nuclei (ECN) were much more distinct than those of veins [48]. The MCA was imaged at five contiguous, non-overlapping segments. For quantitative analysis, the area of 80–100 ECN was determined from the MCA and five branching arteries per animal [49] using ImageJ. Nuclei located at the edge of the vessel or near a branching point were excluded.

### Statistics

GraphPad software was used. A Student's unpaired *t*-test was used to determine differences in ECN area and immunohistochemical staining between groups. Diastolic BP and VEGF levels were analyzed using two-way repeated measures ANOVA. One-way ANOVA was used to compare ECN area across age. Results are expressed as mean ± standard error of the mean.

## Results

### Physiological Parameters

Middle-aged female mice provided with wheels ran on average 9.7±0.3 h/day, almost exclusively during the dark cycle, and covered a distance of 9.8±0.5 km/day. Food intake and body weights were determined three times/week. Food consumption was greater in exercising compared to sedentary mice (sedentary: 2.5±0.1 g/day; exercised: 3.5±0.2 g/day, p<0.0001 two-way repeated measures ANOVA). No difference in body weight between groups was observed across study duration (sedentary: 24.8±0.3 g *vs* exercised: 25.0±0.3 g). Thus, despite increased food intake, weight was maintained in exercising animals.

### Exercise Mitigates Glial Markers of Brain Aging

#### Astrocytes

**Figure 1:**
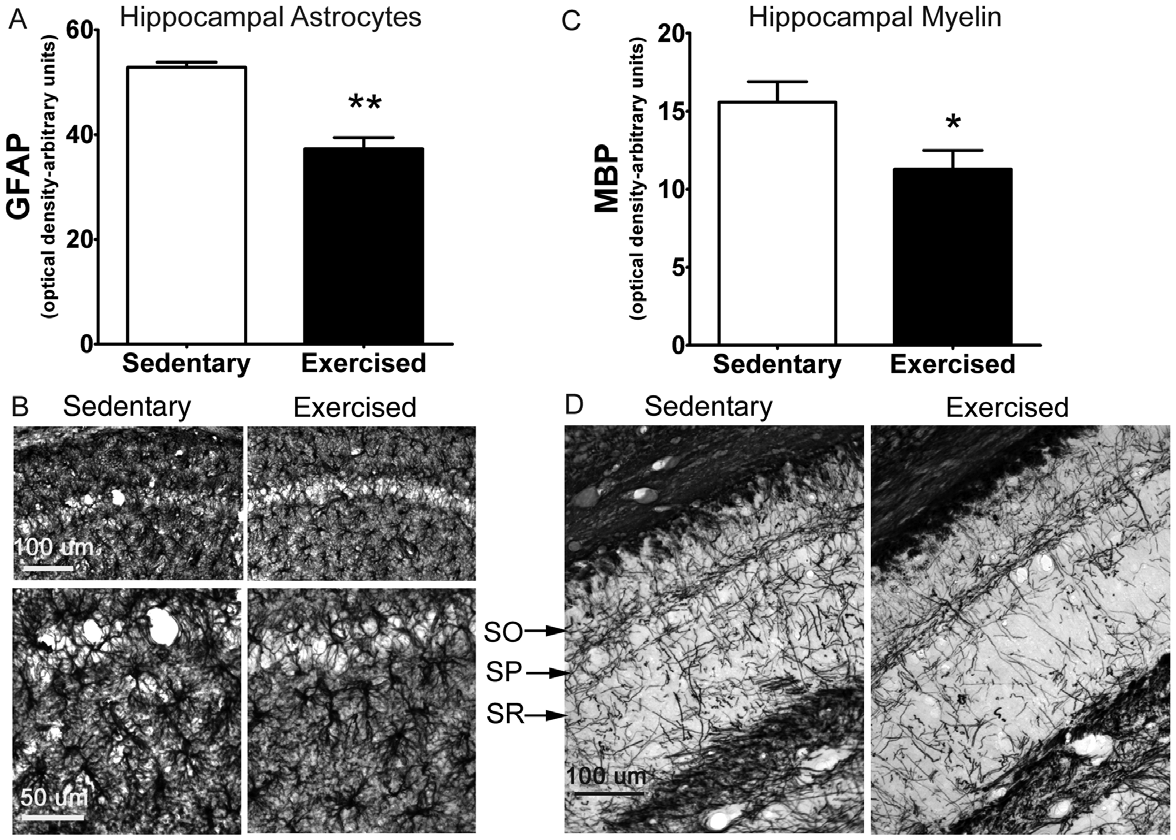
GFAP and MBP immunoreactivity in the CA1 hippocampal region from sedentary and exercised mice. **A and B**, Comparison of GFAP staining in SR from sedentary and exercised mice. Image analysis of mean grey value **(A)** revealed decreased GFAP immunoreactivity in exercised compared to sedentary mice **(B)**. **C and D**, Comparison of MBP staining of SR in sedentary vs. exercised mice. Image analysis of mean grey value **(C)** revealed decreased MBP immunoreactivity in exercised compared to sedentary mice **(D)** SO = stratum oriens, SP = stratum pyramidale, SR = stratum radiatum. *p<0.05, **p<0.0001, *t*-test (n = 7/group).

A well established biomarker of brain aging is the increase in reactive astrocytes which are larger, more stellate and inflammatory in nature [17], [50]. Therefore, we examined the effects of exercise on astrocytes within the stratum radiatum of the hippocampus using an anti-GFAP antibody. Immunostaining within this region was significantly reduced by 30% in exercised mice (p<0.0001, *t*-test) (Fig. 1A, B). In general, astrocytes appeared to have fewer branches, finer processes and were less hypertrophied in exercised animals.

#### Myelin

In prior studies we have shown that aging is associated with an increase in the expression of myelin-related genes and proteins in the hippocampus [24], [27]. We, therefore, examined the effects of exercise on MBP, an abundant protein marker of myelin. The stratum radiatum contains the myelinated axons of the Schaffer collaterals that synapse on dendrites of CA1 pyramidal neurons and were of particular interest because these functional connections are altered with aging [51]. MBP immunoreactivity was reduced in exercised mice by ∼30% (p<0.05, *t*-test) (Fig. 1 C, D).

### Exercise and Vascular Changes in Middle-Aged Mice

#### Peripheral Effects

**Figure 2:**
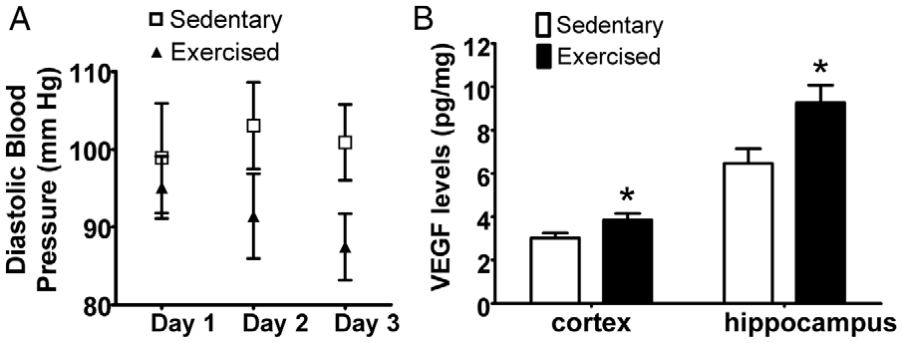
Vascular effects of exercise. A , Exercise reduced diastolic BP in middle-aged mice. Blood pressure was recorded for three consecutive days following two days of acclimatization. Two-way repeated measures ANOVA showed an overall difference in diastolic BP between sedentary and exercised groups (*p*<0.05, n = 10/group). **B**, Exercise increased brain VEGF levels. VEGF ELISA was used to measure levels in cortical and hippocampal tissues. Overall ANOVA is significant for a difference in brain VEGF levels between groups. *p≤0.01, RM ANOVA (n = 7/group).

At five weeks of running, BP was recorded from all animals over three consecutive testing days. An overall main effect of exercise was found on diastolic BP, but not systolic BP or pulse rate. Figure 2A shows that wheel running reduced diastolic BP (p<0.05, two-way repeated measures ANOVA). The effect was largest on the third day raising the possibility that the restraint stress associated with tail-cuff BP testing may have partially obscured effects on the first two days prior to acclimatization [52], [53].

#### CNS Effects

Because VEGF is a prominent marker of angiogenesis [54] and decreases with aging in the hippocampus [50], VEGF protein levels were measured in sedentary and exercised mice. There was an overall main effect of exercise on brain VEGF levels (p≤0.01, two-way repeated measures ANOVA). Compared to sedentary mice, VEGF levels were significantly increased by 27% and 43%, respectively, in the cortex and hippocampus of exercised animals (Fig. 2B).

#### Age-Related Endothelial Changes

**Figure 3:**
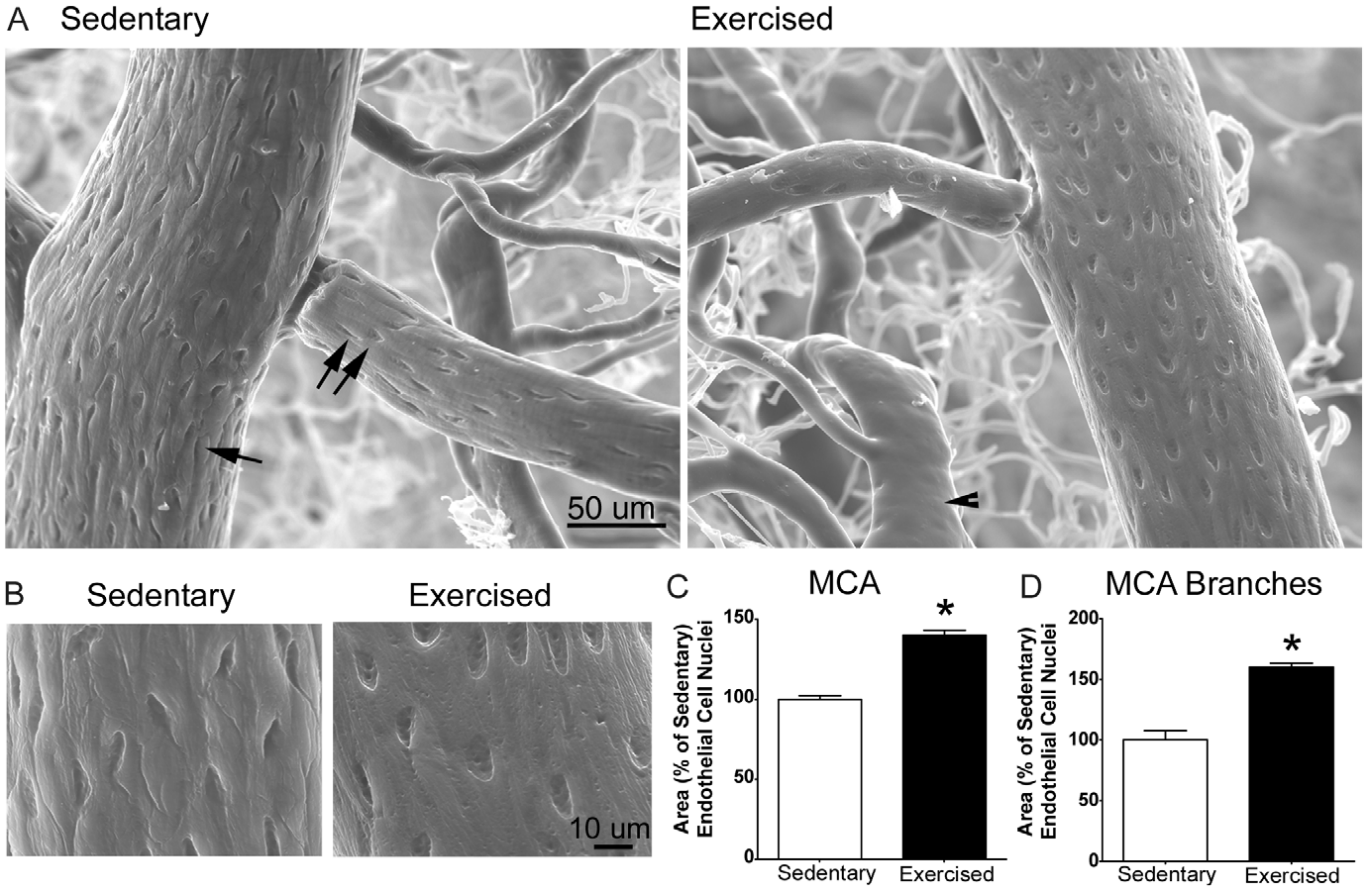
Scanning electron micrographs comparing cerebrovascular microstructure of sedentary and exercised middle-aged mice. A , Low magnification images of the middle cerebral artery (MCA) (single arrow) with branch (double arrow) in a sedentary (left) and exercised (right) mouse. Arteries are distinguished from veins (arrowhead) by the distinct impressions made by their endothelial cell nuclei (ECN). **B**, High magnification images of ECN imprints from the MCA in a sedentary (left) and exercised (right) mouse. **C and D**, Exercise increased the area of ECN in the MCA **(C)** and its associated branches **(D)**. *p<0.005, *t*-test (n = 3/group).

Effects of exercise on the cerebrovascular microstructure were also assessed in a subset of mid-aged sedentary and exercised mice using vascular corrosion casting. Scanning electron micrographs from sedentary and exercised mice show distinct structural differences in the middle cerebral artery (MCA) and associated branches (Fig. 3A). These vessels appeared ragged and irregular in sedentary mice but in exercised mice had a smoother, more uniform structure. Vascular casts also revealed well-defined imprints of the ECN (endothelial cell nuclei) which were quantified as previously described [49]. Exercised mice had ECN areas that were ∼50% greater than sedentary animals (p<0.005, *t*-test; Fig. 3C, D) and were characterized by a more regular, elliptical appearance, whereas sedentary mice had misshapen ECN that appeared compressed and disorganized in orientation (Fig. 3B).

**Figure 4:**
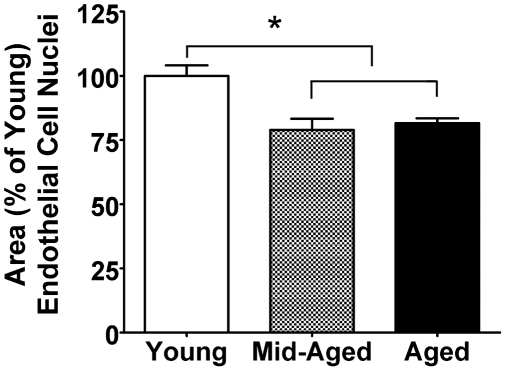
Endothelial cell nuclear area decreased with age. Decreased ECN area in the MCA of middle-aged and aged animals compared to young. *p<0.005, *one-way ANOVA with post-hoc Tukey multiple comparison test,* (n = 6–7/group).

Because the effects of normal aging on cerebrovascular microstructure have not been investigated with casting techniques, we examined ECN area in a separate group of young, mid-aged, and aged mice. Compared to young mice, both older groups of animals had ECN that were more irregular in appearance and significantly smaller by ∼20% (p<0.005, one-way ANOVA and post-hoc Tukey) (Fig. 4). ECN areas were normalized to young values and were 100±4.1% in Young, 78.9±4.4% in Mid-aged, and 81.6±1.9% in Aged. There was no difference in ECN areas between mid-aged and aged mice.

## Discussion

Although multiple studies have shown beneficial effects of exercise on cognitive decline and neuronal function in brain aging, this is among the first to show that exercise also counters several well-established glial markers of brain aging. Similarly, we show that age-related changes in neurovascular morphology and function were reduced with exercise. Specifically, exercise counteracted the aging-dependent glial changes of astrocyte hypertrophy and enhanced myelination and also attenuated aging changes in the morphology of vascular endothelial cells and the expression of VEGF. Thus, our results show that exercise can potentially mitigate progressive age-related changes in several key non-neuronal elements of the brain. Further, we show that these brain processes are still highly responsive to exercise in the midlife age range, consistent with studies showing that cognitive function can benefit from exercise even if initiated at later ages.

### Possible Implications of Exercised-induced Reductions in Astrocyte Hypertrophy/Reactivity in Aging

Astrocytes are the most numerous cells in the brain and play important roles in many aspects of brain function [55], [14]. However, with aging astrocytes hypertrophy and become reactive [22], an effect observed in animals and humans [15]–[21]. While multiple mechanisms may play a role, the cerebrovascular changes typical of aging (e.g., alterations in density, ultrastructure, plasticity) [29] may represent an underappreciated factor that contributes to astrocyte reactivity with age [56]. Through their end-feet contacts with blood vessels, astrocytes serve as conduits to transfer energy metabolites from the circulation to gray and white matter in the brain. Following injury, astrocytes become reactive and increase their arborizations in what is believed to be a compensatory response to facilitate neurovascular coupling and metabolic support to neurons [57]. Therefore, it seems possible that in aging, astrocytes may sense impaired cerebrovascular function and become increasingly reactive over time. Although this change may initially be beneficial, with chronic vascular dysfunction, long-lasting astrocyte reactivity could result and promote deleterious downstream consequences [58]. For example, reactive astrocytes produce high levels of endothelin, a potent vasoconstrictor [59]. Given that astrocytes also mediate local cerebral blood flow [46], endothelin release from reactive astrocytes could potentially exacerbate preexisting age-related changes in blood flow [60]. Reactive astrocytes can also inhibit neurogenesis [61], a key mechanism believed to underlie the cognitive benefits of exercise [1], [13]. Together with studies showing that exercise improves vascular/endothelial function [36], [3], [29], [37], our results of reduced astrocyte hypertrophy with exercise suggest that glial changes in aging may be dependent, at least in part, on age-related vascular dysfunction, and that the process may be reversible with exercise.

### Exercise Attenuates the Midlife Increase in Myelinogenesis

Age-related cognitive decline is characterized by slower processing speeds which may be due to compromised myelin integrity [62], [23], [28]. In prior studies, we observed an increase in myelin-related staining with age and an upregulation of myelinogenic genes and proteins beginning at midlife [24], [27]. Further, studies of myelin ultrastructure at midlife and later ages also show abnormal ballooning and redundant or thickened myelin [23]. Optimal myelination facilitates neural conduction and is maintained by a balance between myelinating and demyelinating processes and either too little or too much myelin impairs conduction [63]. A potential explanation for the increase in midlife myelin is that it results from a shift in the processes that maintain optimal myelination. Further, this shift may reflect the earliest consequences of age-related changes in cerebrovascular function. Here, exercise reduced myelin staining in the hippocampus perhaps by restoring a balance between myelinating and demyelinating processes. As noted above, an exercise-induced improvement in cerebrovascular function may be an underlying mechanism. Support for this notion comes from studies showing that significant myelin pathology can be induced by chronic cerebral hypoperfusion [64], [65].

### Does Exercise Promote Cerebrovascular Fitness in Brain Aging?

Among our observations, the exercise-induced plasticity of the cerebrovasculature at mid-age is notable. Brain aging is characterized by decreases in vascularity and endothelial function, factors which contribute to observed age-related reductions in vascular function [60], [29]. While exercise increases angiogenesis and cerebral perfusion [66], [67], few studies have examined the neurovascular effects of exercise during the mid-age period. VEGF, a prominent marker of angiogenesis [54], decreases with aging in the hippocampus [50]. Further, one of the most consistent gene expression markers of brain aging identified in our microarray studies is a reduction in VEGF [24], [25], [27]. VEGF expression is also reduced in human skeletal muscle with aging, an effect reversed by exercise [68]. The present results show that exercise can increase brain VEGF levels and may promote blood vessel formation in the brain even when initiated with advancing age.

The vascular casts provide a unique view of the cerebrovascular lumen [48] and show distinct structural changes in the endothelium which appear to be age-related (*see below*). The endothelium plays a key role in maintaining an optimal vessel wall and surface irregularities can produce turbulence and other factors that impair cerebral blood flow [69]. In our study, cerebral vessels in exercised mice had a smoother surface, which may facilitate laminar blood flow and make them less prone to thrombogenic events than sedentary vessels. The vascular casts here also revealed larger endothelial cell nuclei (ECN) with exercise. Because a decrease in ECN area is associated with the endothelial dysfunction of hypertension [70], perhaps the larger ECN with exercise reflect improved endothelial function. Further, vascular casts from young, mid-aged and aged animals showed that ECN area decreased in an age-dependent manner. Together, these results suggest that the cerebrovascular endothelium undergoes progressive age-related changes that may result in endothelial dysfunction but which may be reversed by exercise initiated at mid-age. The exercised group also had lower diastolic BP, raising the possibility that changes in peripheral BP may influence structural changes in the cerebrovascular endothelium [36]. Along these lines, it is noteworthy that higher diastolic BP with aging is associated with atrophy of cerebral arterioles, white matter damage, and cognitive impairment [71].

### Conclusion

**Figure 5:**
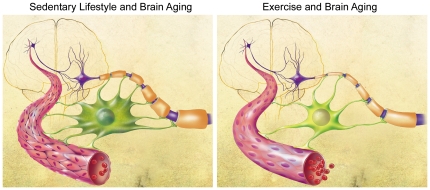
Model of exercise effects on brain aging. *Left Panel*. Endothelial changes associated with aging lead to cerebrovascular dysfunction. Astrocytes become reactive in an attempt to improve neurovascular coupling by increasing their arborizations. With chronic vascular dysfunction, white matter eventually begins to break down, but attempts at remyelination are impeded by hypertrophied and reactive astrocytes. As a result, the myelin becomes disorganized and is functionally compromised. *Right Panel.* Exercise initiated at mid-age promotes endothelial integrity and thereby cerebrovascular health. In turn, these changes ease the burden of the astrocyte in maintaining neurovascular coupling as reflected by a less hypertrophied and reactive astrocyte. Under these conditions, aberrant myelin production and turnover is reduced, and a more optimal myelin structure and function is restored. *Illustration by Tom Dolan, University of Kentucky.*

A potential model that takes these and prior observations into account is shown in Figure 5. Here, chronic hypoperfusion, as a result of aging [29] and a sedentary lifestyle, challenges the astrocyte's role in neurovascular coupling *(left panel)*. Although astrocyte hypertrophy/reactivity may initially represent a compensatory response to preserve metabolic coupling, without improvements in vascular function, chronic reactivity may set in. Reactive astrocytes can acquire maladaptive functions and may impair vascular integrity even further. Myelin begins to succumb to changes in perfusion and optimal myelination is harder to maintain, thwarted by reactive astrocytes which are increasingly unable to provide adequate metabolic support or secrete factors which support myelin [72], [44], [73], [47]. As a result, myelin-related genes and proteins upregulate [24], [25], [27] to offset these degenerative processes but the response is disordered and may result in thickened myelin, among other abnormalities [23], [27]. Together, such events may contribute to unhealthy brain aging and cognitive decline.

On the other hand, exercise in aging improves (or maintains) cerebrovascular function and eases the astrocyte's burden, thus restoring normal neurovascular coupling (Fig. 5, *right panel* *)*. Astrocyte reactivity and its negative consequences are reduced and the myelin phenotype appears younger. While alternative models could account for our results, the observed exercise-induced changes may underlie, at least in part, the improvements in memory observed in aging individuals that undertake an exercise regimen [2], [4]–[6], [8].

Certainly, the present studies do not preclude the contribution of other known mechanisms associated with the benefits of exercise (e.g., decreased inflammation and oxidative stress, increased neurogenesis and growth factor production) [1]–[3], [9], [13], [7]. Further studies, however, are required to better understand mechanisms underlying the benefits of exercise in brain aging and the relationship between neuronal and non-neuronal elements. Nonetheless, our studies provide some additional insight and suggest that astrocytes may play an important role. Thus, vascular health and myelin integrity in aging may be related through a common interaction with astrocytes. Although we did not compare genders, we used a female animal model [74] and our results may have important implications for aging women [6] especially in light of findings that certain risk factors (e.g., elevated blood pressure) may contribute more to “vascular aging” in women than men [34], [35]. Nevertheless, exercise benefits both genders [2], [4]–[6], [75] and these studies show that an exercise regimen implemented at mid-age can reverse markers of unhealthy brain aging. Given recent projections of a dramatic rise in the elderly population [76], along with the prevalence of cerebrovascular disease [69], interventions that target vascular function [77] and thereby astrocyte reactivity may decrease the burden of unhealthy brain aging and associated cognitive decline.
